# The impact of insulin on chemotherapeutic sensitivity to 5-fluorouracil in gastric cancer cell lines SGC7901, MKN45 and MKN28

**DOI:** 10.1186/s13046-015-0151-8

**Published:** 2015-06-18

**Authors:** Zhao Wei, Li Liang, Liu Junsong, Chen Rui, Chang Shuai, Qiu Guanglin, He Shicai, Wang Zexing, Wang Jin, Che Xiangming, Wang Shufeng

**Affiliations:** Department of General Surgery, The First Affiliated Hospital, Xi’an Jiaotong University, Xi’an, Shaanxi 710061 P.R.China; Health science center, Xi’an Jiaotong University, Xi’an, Shaanxi 710061 P.R.China; Department of Neonatal Surgery, The Children’s Hospital, Zhejiang University School of Medicine, Hangzhou, Zhejiang 310003 P.R.China

**Keywords:** Gastric cancer, Chemoresistance, 5-fluorouracil, Insulin, P-glycoprotein

## Abstract

**Background:**

The role of insulin in the pathogenesis of cancer has been increasingly emphasized because of the high incidence of obesity and metabolic syndrome and their correlated complication including cancer. This study aimed to explore the impact of insulin on chemoresistance to 5-fluorouracil in gastric cancer and the possible mechanisms.

**Methods:**

Tissue samples of gastric cancer and adjacent normal gastric mucosa from patients with or without obesity were performed immunohistochemical staining for P-glycoprotein. The follow-up was done after the surgical treatment. The effect of insulin on chemotherapeutic sensitivity of the three gastric cancer cell lines to 5-fluorouracil was evaluated by pre-incubation with insulin before administration of 5-fluorouracil. The expression of P-glycoprotein was determined by Western blotting.

**Results:**

P-glycoprotein were overexpressed in tissues from patients who suffered gastric cancer and were higher in those simultaneously suffered gastric cancer and obesity. Addition of 1 μM insulin remarkably promoted the proliferation of SGC7901, MKN45 and MKN28 cells and decreased the cytotoxicity of 5-fluorouracil. In addition, the expression of P-glycoprotein was upregulated in SGC7901, MKN45 and MKN28 cells.

**Conclusion:**

Insulin improved the proliferation of gastric cancer cell lines and contributed to chemoresistance of gastric cancer cells to 5-fluorouracil which is likely to involve upregulation of P-glycoprotein.

## Introduction

Obesity is one of the most prevailing diseases in the Western countries and increases rapidly in developing countries in recent years [1]. The complications of obesity have been widely studied due to the large obese population. Cancer was not thought to be one of the mortal complications of obesity until recent decades. It was reported that up to 20% of all cancers could be attributed to obesity [2,3]. The potential mechanisms underlying increased cancer risk in the obese relate to multiple molecular and metabolic alterations include elevated hormones and growth factors [4]. The altered systemic and local environment that occurs as a consequence of obesity can also create the potential for unfavorable responses to chemotherapy [5]. For instance, obesity was reported to impair responses to carboplatin in breast cancer [6,7] and to bevacizumab-based therapy of colon cancer [8]. In animal study of human basal-like breast cancer, diet-induced obesity was found to induce resistance to several chemotherapeutic agents [9]. The detailed mechanisms still are not clear, but the metabolic dysregulations occurred in obesity were paid more attention recently in consideration of numerous related experimental results. Chen, et al. found that high levels of insulin conferred resistance to oxaliplatin in colon cancer cell lines [10]. In addition, chemoresistant cells displayed an increased proliferative response to insulin [11]. These results demonstrate that the effects of obesity-related metabolic disorder on chemoresistance at least partly are exerted by insulin.

P-glycoprotein in cancer research is widely known for its role of causing multidrug resistance by its drug efflux effect dependent on ATP. The hydrophilic regions of P-glycoprotein contain nucleotide-binding sites and display the characteristic domains of the ATP-binding cassette (ABC), which are responsible for its ATPase activity enabling the pumping of multiple substrates including antibiotics and antitumor drugs against the concentration gradient [12].

Although the clinical and experimental studies that link P-glycoprotein to obesity-associated drug resistance are absent, the connection between insulin and P-glycoprotein has been established. For example, Animal study revealed that P-glycoprotein expression decreased in insulin-deficient mice [13]. Addition of insulin to the insulin-deficient diabetic rat normalized the impaired function and expression of P-glycoprotein in brain microvessel endothelial cells [14,15]. In cancer researches, some experimental studies involving insulin associated signaling pathways pointed out the possible synergism between insulin and P-glycoprotein in cancer chemoresistance. These studies showed that the role of PI3K/mTOR signaling and P-glycoprotein in resistance to doxorubicin in hepatoma cells [16] and that MAPK signaling is involved in P-glycoprotein overexpression in chemoresistant cancer cells [17]. The link between insulin and P-glycoprotein in contribution to cancer chemoresistance may also correlate with β-catenin [4,18].

Though plenty of evidence has pointed to the potential relationship of insulin and P-glycoprotein and chemoresistance, the direct contacts of insulin with cancer chemoresistance still are paucity, especially in the research of gastric cancer in spite of previous epidemiological findings that gastric cancer was associated with obesity [19,20] and experimental findings that linked PI3K/AKT or MAPK signaling pathways to gastric cancer [21,22]. Chemotherapeutic regimens containing 5-fluorouracil is the main treatment of gastric cancer besides surgery. Given the large population of obesity and the discounted efficacy of chemotherapy in gastric cancer treatment of obese patients, to investigate whether insulin lead to resistance to 5-fluorouracil has significant clinical value.

In our study, we try to explore the direct influence of insulin on the chemotherapeutic sensitivity of three gastric cancer cell lines to 5-fluorouracil. We do not attempt to elucidate the detailed mechanisms of insulin-induced drug resistance but to provide an in vitro clue which may be beneficial for further research on gastric cancer and clinical treatment.

## Materials and methods

### Tissue specimens

Tissue samples of gastric cancer and adjacent normal gastric mucosa (5–10 cm apart from the primary tumors) from the same patient were collected from 41 patients of gastric cancer (18 gastric cancer without obesity vs. 23 gastric cancer & obesity; the obesity was defined as BMI > 30 or waist-to-hip ratio >0.9 (male) or >0.85 (female) according to the WHO criteria of obesity made in 1999 [23]) undergoing surgical resection at the First Affiliated Hospital, Xi’an Jiaotong University(Xi’an, China) from January, 2000 to December, 2004. All tissues were snapfrozen in liquid nitrogen and stored at −80°C. Each case was reviewed by 2 experienced pathologists. All patients had received neither chemotherapy nor radiation therapy before tumor resection. The follow-up was done after the surgical treatment. The study protocol was approved by the local ethics committee, and written, informed consent was obtained from each patient.

### Immunohistochemisty

Immunohistochemical staining for P-glycoprotein was performed on formalin-fixed paraffin-embedded tissue sections of gastric cancer using the streptavidin-peroxidase method. Primary antibody of P-glycoprotein was purchased from Biosynthesis biotechnology Co., LTD (Beijing, China).The results were evaluated by counting 100 cells per field in 10 random fields under high-magnification microscopy (×400, Olympus BX53 [Olympus, Tokyo, Japan]). Positive staining was defined as ≥ 25% staining; Negative staining as <25% staining [24].

### Cell lines

Human gastric cancer cell lines SGC7901, MKN45 and MKN28 and the immortalized normal gastric mucosa cell line GES which was used as control were obtained from the Fourth Military Medical University, China. These cell lines have been tested and authenticated. These cell lines were maintained in RPMI-1640 medium supplemented with 10% fetal bovine serum at 37°C and 5% CO2.

### Treatment of gastric cancer cell lines with insulin and 5-fluorouracil

Three gastric cancer cell lines were cultured in medium without serum for 24 h before treatment, respectively. Insulin (Sigma Aldrich) was added into serum free RPMI1640 medium at different concentrations before replacing the former medium with or without administration of 5-fluorouracil (Sigma Aldrich).

### Cell viability assay

Cell viability was determined by Cell Counting Kit-8 (CK04, DOJINDO Laboratories, Japan) according to the kit manual. Cells were seeded in 96-well plates at a density of 1000–10,000 cells per well in RPMI-1640 medium with 10% FBS with or without added agents. After 24, 48 or 72 hours culture, 10 μl of CCK-8 was added to each well to incubate for 1 hour. Then the mean OD values were examined in 450 nm wavelength by microplate reader (iMark 680, Bio-rad, US).

### Western blotting

Cells were washed twice with cold PBS and lysed in 150 μl lysis buffer. Proteins were separated by electrophoresis in 10 well SDS-PAGE gels by a Bio-Rad apparatus. The proteins were then transferred into polyvinylidene difluoride membranes. The membranes were blocked at room temperature with 5% defatted milk solution for 1 hour. After that, the membranes were incubated at 4°C overnight with addition of rabbit anti-MDR1 antibody (bs-0563R, Beijing Biosynthesis Biotechnology Col., LTD, China) at 1:500 dilution. After four times of 10 min wash with TBST, the membranes were incubated at 37°C for 2 hours with horseradish peroxidase conjugated goat anti-rabbit antibody at dilution 1:2000. The membranes were then exposed to Fujifilm after incubating with ECL (12142012, CWBiotech, China) for 1 minute. Densitometry analysis was performed by using Bio-rad quantity one software.

### Statistics

Quantitative results were expressed as mean ± standard error. Multiple comparisons were performed by 1-way analysis of variance followed by the Dunnet t post-test. Differences between experimental groups with P values less than .05 were considered significant. All the statistical analysis were done by using SPSS (version 21, Chicago, IL, USA). Two-sided P-values < 0.05 were considered to be statistically significant.

## Results

### P-glycoprotein expression in gastric cancer tissues

**Figure 1:**
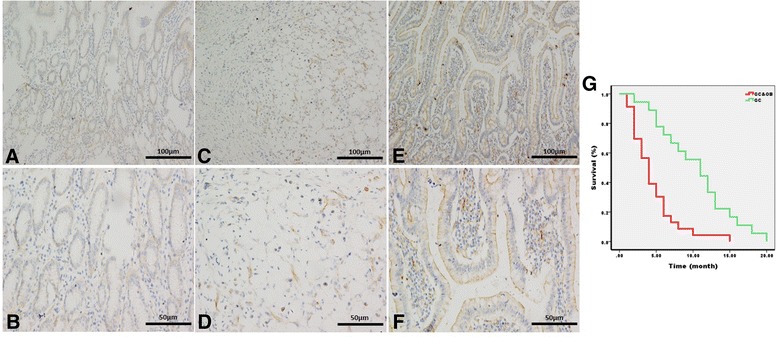
**Immunohistochemical staining in adjacent normal mucosa and gastric cancer tissues. A** and **B** were adjacent normal mucosa while **C,D,E** and **F** were gastric cancer tissues within which E and F were from patient who simultaneously suffered gastric cancer and obesity. The original tissues of **C—F** were evaluated to be grade I tumor according to the 3rd Japanese classification of gastric carcinoma. **C** and **D** were considered to be diffuse type in Lauren Classification of gastric cancer whereas **E** and **F** were intestinal type. The follow-up was showed by graph **G** within which the red foldline stands for gastric cancer & obesity group and the green one represents gastric cancer group.

P-glycoprotein expression in gastric cancer was determined by immunohistochemical staining. P-glycoprotein expression was observed as brown-yellow particles in the cell membrane and cytoplasm (Figure 1). The rate of positive P-glycoprotein expression in those patients with gastric cancer and obesity was 94.4% (17/18) compared with 60.9% (14/23) in those with only gastric cancer as controls (p < 0.05). The follow-up showed a longer survival time of those nonobese patients with gastric cancer than those suffered simultaneously gastric cancer and obesity (gastric cancer 11 ± 2.108 vs. gastric cancer & obesity 4 ± 0.585, *Log Rank = 14.694, p < 0.05*).

### Insulin promoted cell proliferation of gastric cancer cells

**Figure 2:**
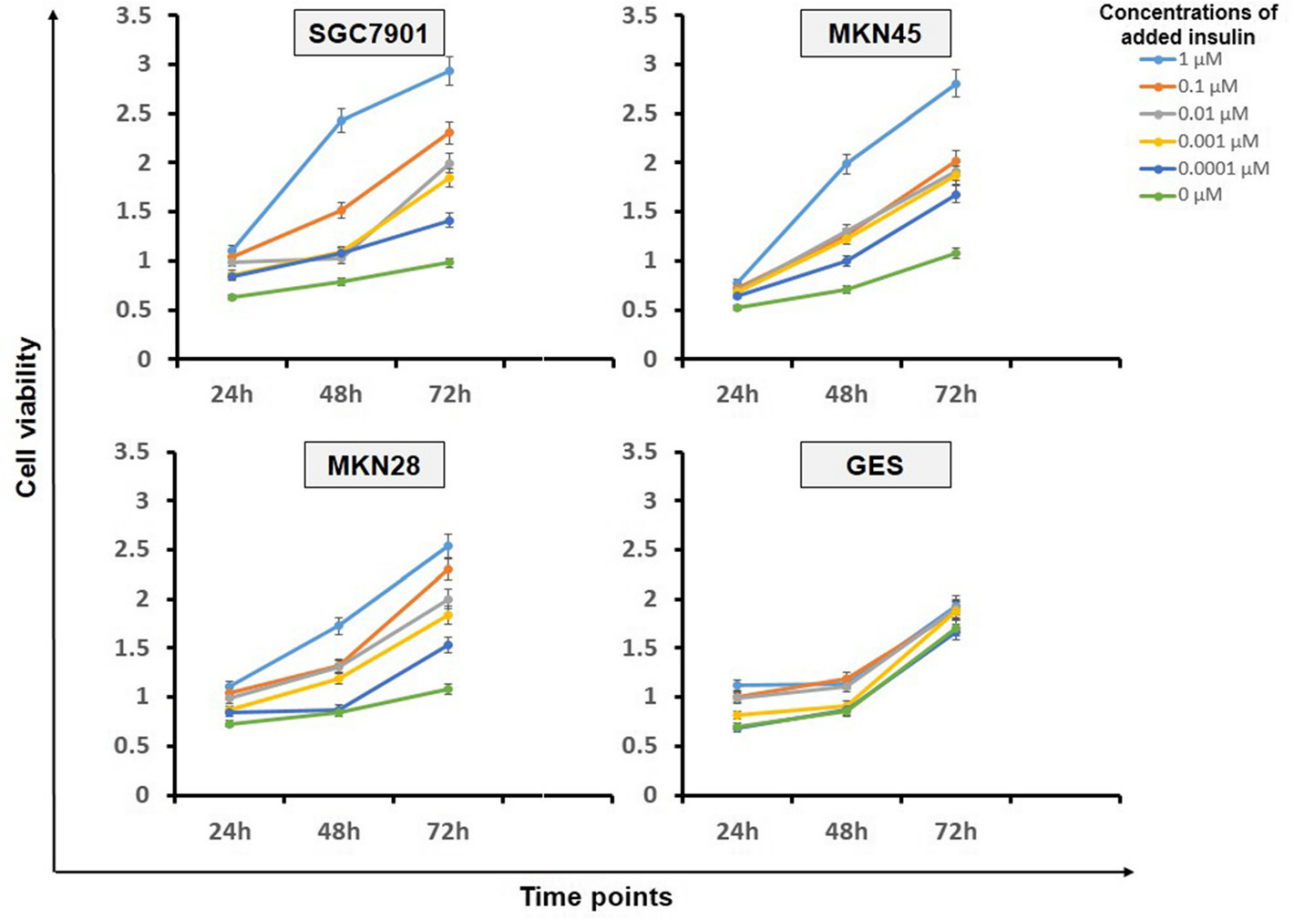
**Proliferation of gastric cancer cell lines with administration of different concentrations of insulin.** The diagram shows the proliferation rate of SGC7901, MKN45, MKN28 cells and controlled GES cells respectively in different time points after administration of different concentrations of insulin.

In our experiment, we found that the proliferation of different gastric cancer cell lines was remarkably accelerated by high level of insulin compared with controlled GES cells (Figure 2). By adding a high concentration of insulin (1 μM), gastric cancer cells proliferated much faster than under a normal insulin concentration of 0.0001 μM whereas the GES cells did not show obvious acceleration of proliferation.

### 5-fluorouracil caused dose- and time-dependent cytotoxicity on cancer cells

**Figure 3:**
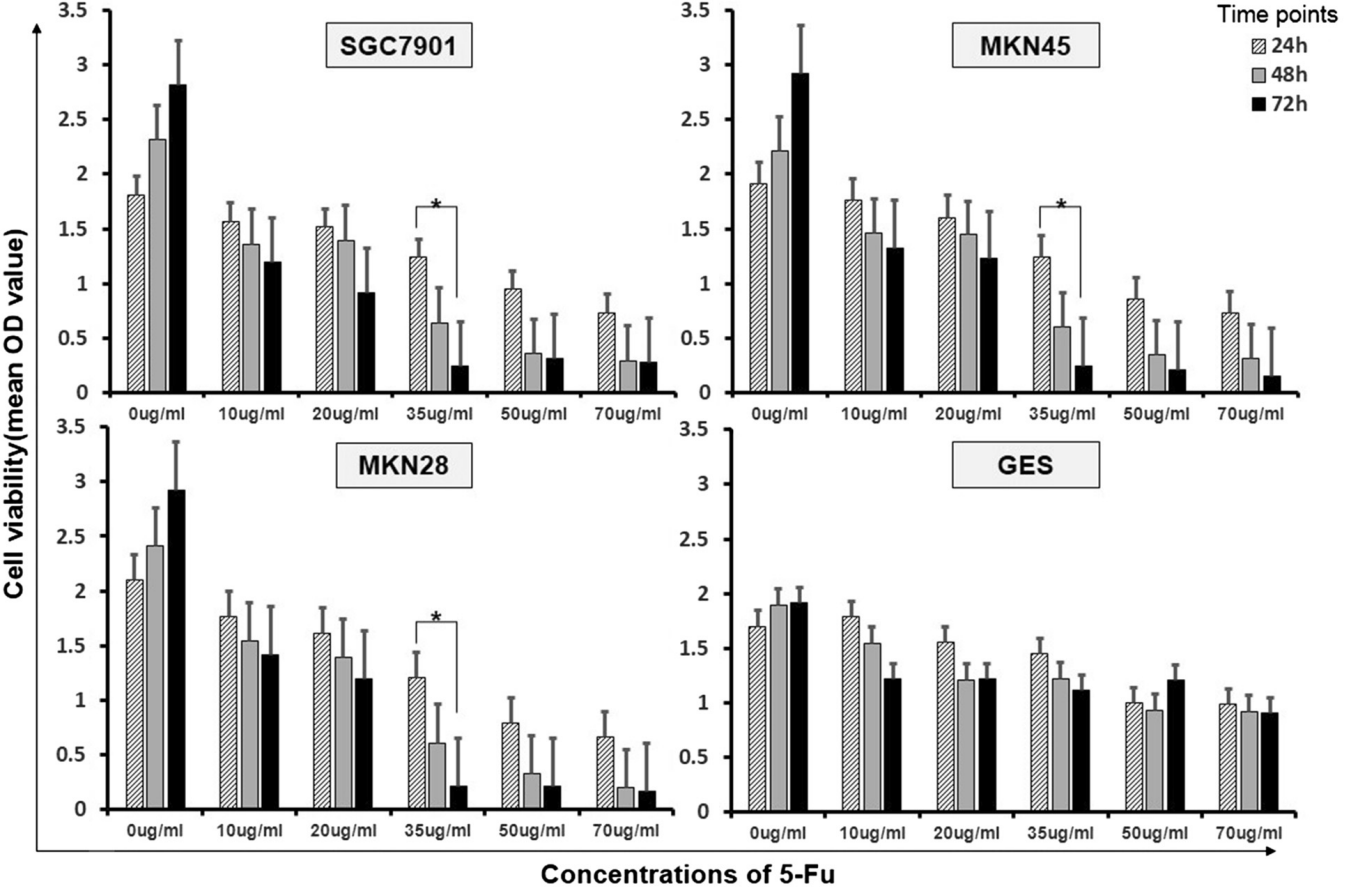
**Cell viability of gastric cancer cell lines with administration of different concentrations of 5-fluorouracil.** The diagram shows the proliferation rate of SGC7901, MKN45, MKN28 cells and compared with GES cells in different time points after administration of different concentrations of 5-Fu with normal insulin.

The effect of 5-fluorouracil on gastric cancer cell viability was measured by CCK-8 assay (Figure 3). Gastric cancer cells and controlled cells were treated with different concentrations of 5-fluorouracil without addition of insulin. After treatment, the growth of gastric cancer cells was inhibited obviously in comparison with GES cells. Inhibition was time and concentration dependent in gastric cancer cells within which statistical difference was presented under the concentration of 35ug/ml between 24 h and 72 h.

### High Insulin induced chemoresistance of gastric cancer cells to 5-fluorouracil

**Figure 4:**
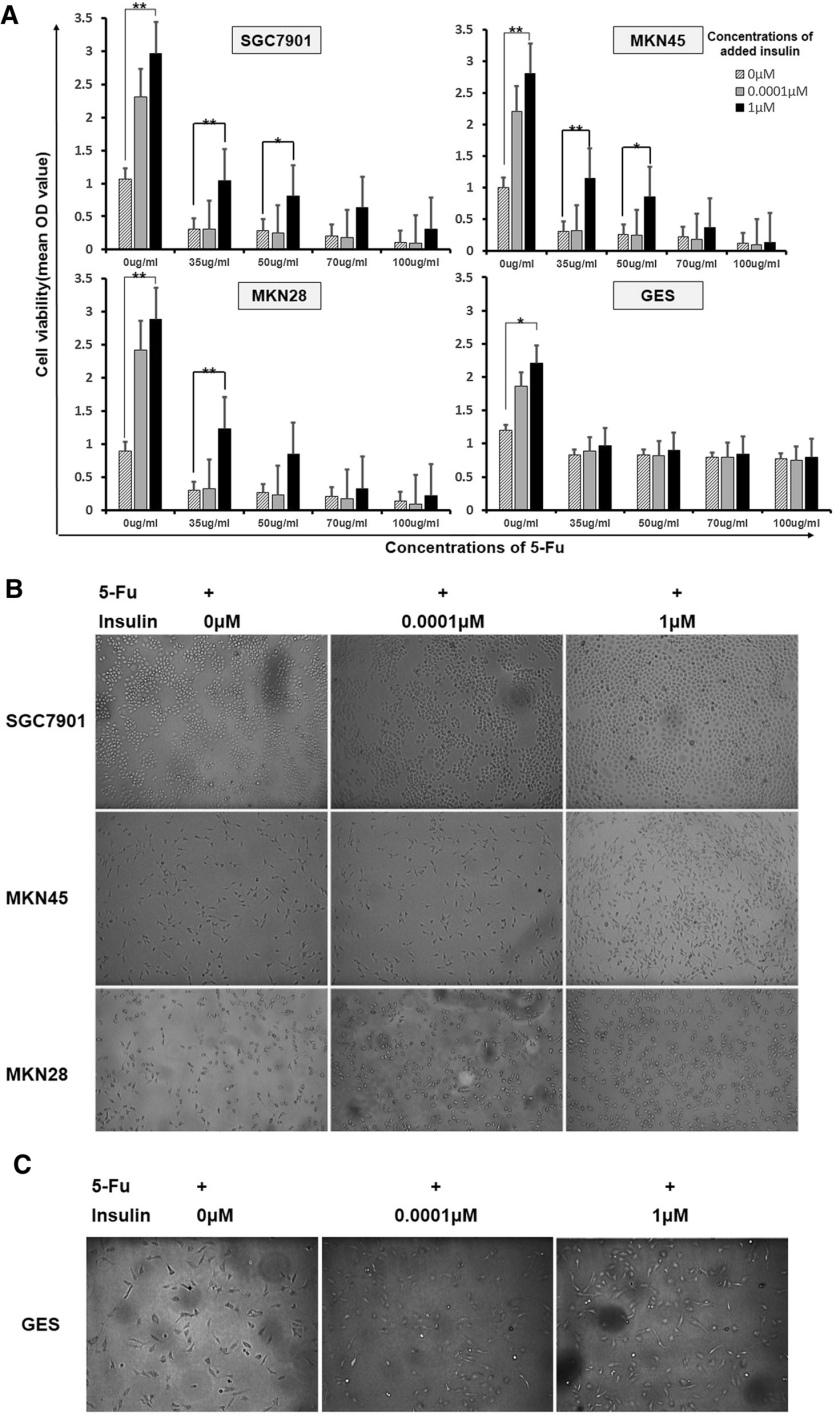
**Cell viability of gastric cancer cell lines with administration of different concentrations of 5-fluorouracil and insulin.** Graph **A** show the proliferation rate of SGC7901, MKN45, MKN28 cells in contrast to GES cells in 48 hours after administration of different concentrations of 5-Fu with high, normal or without insulin. **B** and **C** shows the photographs of three gastric cancer cell lines and the control GES cells in the conditions of 5-fluorouracil and different concentrations of insulin at 72 hours.

Since results above showed that high level of insulin promoted gastric cancer cells growth and 5-fluorouracil was therapeutically effective to gastric cancer cells, we then examined whether high insulin induced chemoresistance to 5-fluorouracil in gastric cancer cells. The number of live cells was measured by CCK-8 assay as described above. In the presence of 1 μM of insulin, the toxic effect of 5-fluorouracil on gastric cancer cells was apparently compromised and cell number was much more after treatment than with low concentration of insulin or without insulin. This phenomenon did not disappear until the concentration of 5-fluorouracil was added up to 100 μg/ml (Figure 4).

### The expression of P-glycoprotein in gastric cancer cells was upregulated with the administration of insulin when treated with 5-fluorouracil

**Figure 5:**
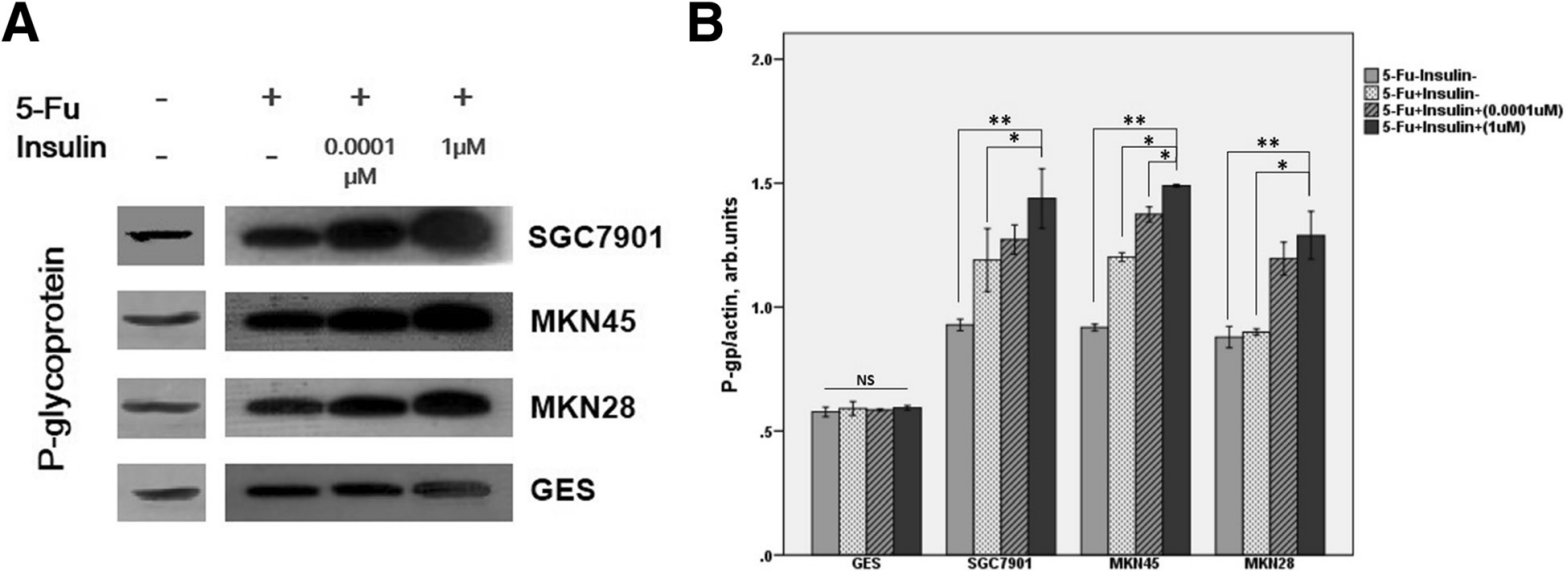
**Immunoblotting of P-gp expression in three gastric cancer cell lines in comparison with GES cell line under the condition of adding different concentrations of insulin and 5-fluorouracil at 72 h. A** is the blotting of P-gp and **B** is the densitometry.

We used Western-blot analyses to investigate the expression of P-glycoprotein in different gastric cancer cell lines with or without administration of insulin and 5-fluorouracil. As shown in Figure 5, P-glycoprotein expression increased in gastric cancer cells after treatment of 5-fluorouracil compared with GES cells. With the presence of insulin, the level of P-glycoprotein was highly upregulated in gastric cancer cells in contrast to the nonobvious alteration in GES cells. These densitometry analysis was in accordance with immunoblotting (Figure 5).

## Discussion

Chemotherapy is the major method for cancer treatment besides surgery, but the efficacy of chemotherapy is often limited in clinic, the main predicament of which is drug resistance, especially multidrug resistance (MDR). Resistance to chemotherapeutics can be divided into two categories: intrinsic or acquired. Both patterns of chemoresistance involves the increased expression of members of the ATP binding cassette (ABC) transporter superfamily [25,26]. P-glycoprotein (namely ABCB1 or MDR1), encoded by MDR1 gene, is one of the most widely characterized MDR transporters in ABC family. It can be expressed in almost all normal tissues. P-glycoprotein can also be genetically overexpressed in tumors (thus causing intrinsic drug resistance) or induced after chemotherapy (thus also resulting in the acquired development of MDR). P-glycoprotein has long been studied in cancer researches. A number of studies implicated that P-glycoprotein may contribute to resistance to antimetabolites, topoisomerase inhibitors and microtubule poisons [27-29]. The clinical relevance between P-glycoprotein and cancer chemoresistanc and prognosis has been evaluated in various tumors such as breast carcinoma [30], acute nonlymphoblastic leukemia cells [31], acute myloid leukemia [32], osteosarcomas [33]. In study of gastric cancer, H-Y Lim, et al. statistically associated the expression of P-glycoprotein in gastric cancer patients with the resistance of 5-fluorouracil and doxorubicin and poor prognosis via immunohistochemical staining [34]. In our study, the results of P-glycoprotein staining in human tissues showed higher expression in cancer than normal tissues. This was in accordance with the results of H-Y Lim.

As we mentioned above, obesity and insulin may contribute to the initiation and progression of a variety of tumors and are linked to low response to antitumor drugs. In our study, we administered insulin and 5-fluorouracil to cultured gastric cancer cell lines SGC7901, MKN45 and MKN28 in comparison with immortalized normal gastric mucosa cell line GES aiming to elucidate the effect of different levels of insulin on the proliferation and chemosensitivity of gastric cancer cells. We found that insulin could significantly promote the proliferation of gastric cancer cells compared with control mucosa cells. When added 5-fluorouracil, the three gastric cancer cell lines showed decreased chemotherapeutic responses when simultaneously administered insulin while the cell viability. This finding is coincident with Chen’s study in colon cancer cell lines [10].

Though the knowledge about the relationship of obesity, insulin and cancer increases extensively, but the applications of experimental findings to clinic are scarce with regard to the complicated changes of metabolism in obesity and the vital functions of insulin in body. Moreover the direct impact of insulin on cancer chemoresistance is absent. In our follow-up study, we also found that the prognosis of obese cancer patients were poor. This was consistent with previous studies. In our study, we also found that the insulin-induced drug resistance to 5-fluorouracil correlated with the augment of P-glycoprotein in gastric cancer. In the experiments, we found that the expression of P-glycoprotein was obviously increased in gastric cancer tissues and with much higher expression in tissues from gastric cancer coupled with obesity. In vitro study of gastric cancer cell lines also showed higher P-glycoprotein under the condition of insulin, especially high insulin, than without insulin in treatment of 5-fluorouracil. The result implicated that insulin may cause the change of P-glycoprotein to impact the proliferation and chemosensitivity of gastric cancer cells. It is likely to have important clinical significance taking the increased population of gastric cancer coupled with obesity into consideration. In the past years, gastric cancer coupled with obesity was in the same managements as gastric cancer without obesity despite the limited efficacy of chemotherapy. If insulin-induced decreased sensitivity to antitumor drugs can be ascribed to P-glycoprotein, the reversal of P-glycoprotein-mediated resistance is likely to work in the treatment of those obese patients who are not sensitive to conventional anti-cancer regimens. The inhibitor of P-glycoprotein was previously used to improve the penetration in physiological barriers and the cytotoxic effect of cytotoxic drugs [35,36]. In obese cancer patients, it may function via both improving the penetration of drugs to lesions or upregulate the sensitivity to anti-cancer drugs.

Our study was merely a superficial observation of the contact of insulin and P-glycoprotein in gastric cancer with obesity. The further investigation on the detailed mechanisms of their influence on cancer chemoresistance and the discovery of its clinical therapeutic significance still have a long way.
